# Comparison of the genetic basis of biofilm formation between *Salmonella* Typhimurium and *Escherichia coli*

**DOI:** 10.1099/mgen.0.000885

**Published:** 2022-11-03

**Authors:** Emma R. Holden, Muhammad Yasir, A. Keith Turner, Ian G. Charles, Mark A. Webber

**Affiliations:** ^1^​ Quadram Institute Bioscience, Norwich Research Park, Norwich, Norfolk, NR4 7UQ, UK; ^2^​ Norwich Medical School, University of East Anglia, Norwich Research Park, Norwich, Norfolk, NR4 7TJ, UK

**Keywords:** Functional genomics, *maoP*, *nuo*, TraDIS, *tomB*, transposon mutagenesis

## Abstract

Most bacteria can form biofilms, which typically have a life cycle from cells initially attaching to a surface before aggregation and growth produces biomass and an extracellular matrix before finally cells disperse. To maximize fitness at each stage of this life cycle and given the different events taking place within a biofilm, temporal regulation of gene expression is essential. We recently described the genes required for optimal fitness over time during biofilm formation in *Escherichia coli* using a massively parallel transposon mutagenesis approach called TraDIS-*Xpress*. We have now repeated this study in *Salmonella enterica* serovar Typhimurium to determine the similarities and differences in biofilm formation through time between these species. A core set of pathways involved in biofilm formation in both species included matrix production, nucleotide biosynthesis, flagella assembly and LPS biosynthesis. We also identified several differences between the species, including a divergent impact of the antitoxin TomB on biofilm formation in each species. We observed deletion of *tomB* to be detrimental throughout the development of the *E. coli* biofilms but increased biofilm biomass in *S*. Typhimurium. We also found a more pronounced role for genes involved in respiration, specifically the electron transport chain, on the fitness of mature biofilms in *S*. Typhimurium than in *E. coli* and this was linked to matrix production. This work deepens understanding of the core requirements for biofilm formation in the Enterobacteriaceae whilst also identifying some genes with specialised roles in biofilm formation in each species.

## Data Summary

Sequence data supporting the analysis in this study has been deposited in ArrayExpress under the accession number E-MTAB-11765 for the *S*. Typhimurium data and E-MTAB-9873 for the *E. coli* data. The authors confirm all supporting data, code and protocols have been provided within the article or through supplementary data files.

**Impact Statement**

Bacterial biofilms cause persistent infections that are difficult to treat with antibiotics. Understanding the genes needed for biofilm formation is important to develop ways to treat these infections effectively. This study used the transposon mutagenesis approach TraDIS-*Xpress* to identify the genes and pathways that affect biofilm development in two important related foodborne pathogens: *Escherichia coli* and *Salmonella enterica* serovar Typhimurium. Genes important in biofilm formation in both species had roles in matrix production, nucleotide biosynthesis, flagella assembly and LPS biosynthesis. Some genes had divergent roles in the biofilm in each species, including *tomB*, which was beneficial to biofilm formation in *E. coli* and detrimental to *S*. Typhimurium. We identified a novel role in biofilm formation for 21 genes in *S*. Typhimurium. This work furthers understanding of the conserved requirements for biofilm formation in Enterobacteriaceae, which could be used to identify biomarkers or targets for biofilm therapeutics.

## Introduction

Bacteria mostly exist in aggregated communities called biofilms. Biofilms complete a lifecycle where free-swimming planktonic cells attach to a surface, become sessile, aggregate, grow and produce a structured community before regaining motility to disperse and colonize new surfaces [1–3]. At each stage of this life cycle, controlled gene expression is required to maximize fitness to meet new challenges. The full complement of genes needed for biofilm formation is not fully understood, and the regulatory elements controlling expression of necessary genes at the appropriate part of the life cycle have not been fully described [4].

Large-scale transposon mutagenesis is a high-throughput and highly accurate tool to link genotype to phenotype, allowing identification of genes that have a significant impact on fitness under a given condition. We recently used TraDIS-*Xpress* to describe the genes required for biofilm formation in *Escherichia coli* over time [5]. TraDIS-*Xpress* incorporates an outwards-transcribing inducible promoter within the transposon, which facilitates altered expression of genes immediately adjacent to transposon insertions. This allows both inactivation and expression changes of genes to be assayed for fitness impacts, which allows both dispensable and essential genes to be studied (where inactivation is not viable) in any condition. Using this approach, we found *E. coli* genes involved in adhesion and motility were beneficial at the beginning of the life cycle, and matrix production, purine biosynthesis and solute transport were only important as the biofilm matured. This study shed light on the requirements for biofilm formation through time and identified roles for five genes that had previously not been implicated in biofilm formation [5].

We wanted to compare the results from our previous study with those for another, related organism widely studied for its ability to form a biofilm: *Salmonella enterica* serovar Typhimurium. Although the two species share a common ancestry and many shared genes they exhibit different lifestyles and are estimated to have diverged approximately 140 million years ago [6, 7]. Therefore, we hoped comparison of the genes involved in biofilm formation in these two related, but different species would begin to reveal genes required for biofilm formation in each and begin to establish a set of core genes needed for biofilm formation in the *Enterobacteriaceae *.

Comparing biofilm development between *S*. Typhimurium and *E. coli* found pathways with conserved importance in both species, including matrix production, nucleotide biosynthesis, flagella assembly and LPS biosynthesis. Differences were observed between the species with genes involved in DNA housekeeping, *dam* and *maoP*, having a greater effect on the fitness of *E. coli* biofilms, and genes involved in respiration affecting *S*. Typhimurium biofilm fitness to a greater degree than *E. coli *. Expression of *tomB* had an opposite effect in each species; its deletion was detrimental to *E. coli* biofilms and beneficial in *S*. Typhimurium.

## Results

### Core pathways involved in biofilm formation in both *E. coli* and *S*. Typhimurium include adhesins, nucleic acid synthesis and global gene regulation

**Fig. 1.**
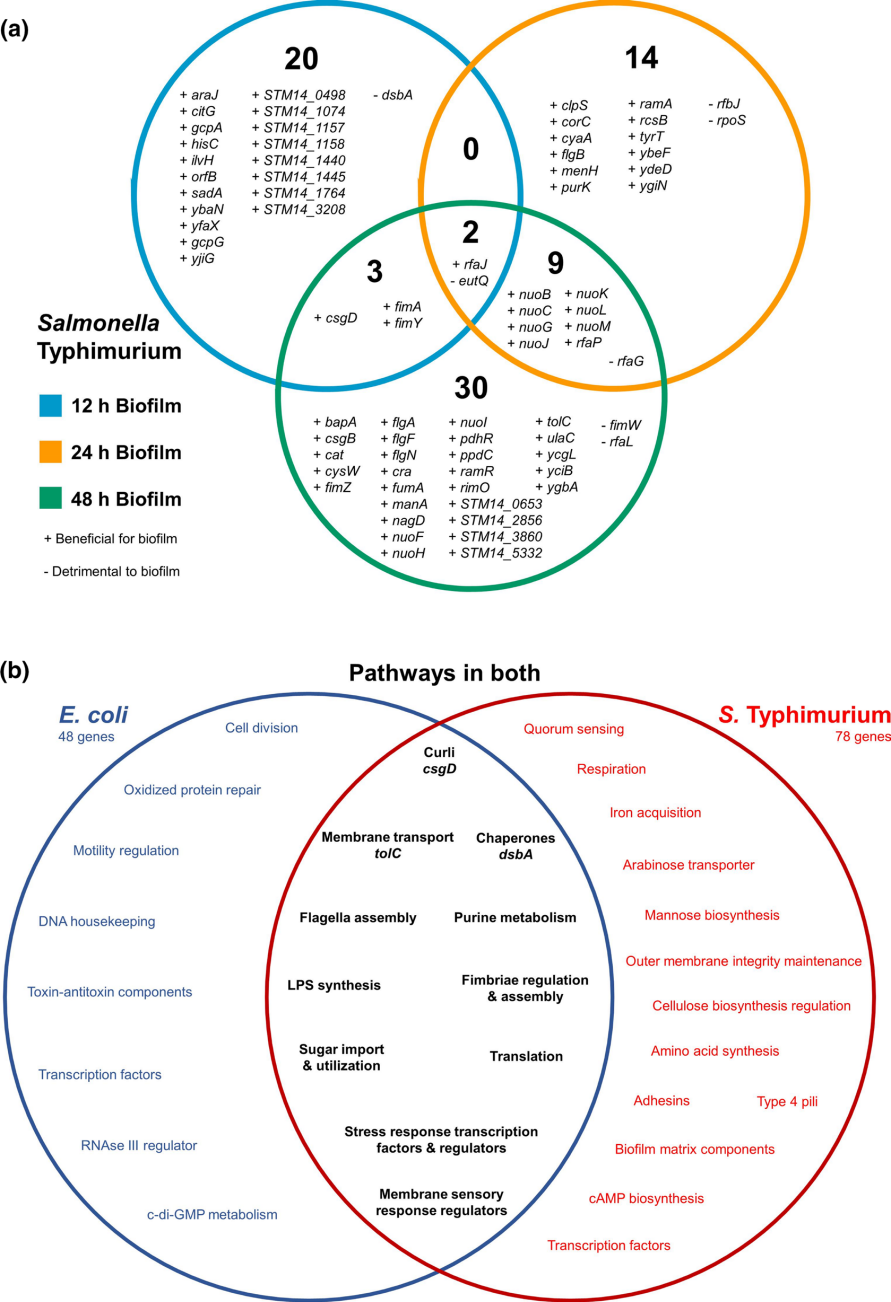
(a) Genes identified by TraDIS-*Xpress* to affect biofilm fitness in *S*. Typhimurium after 12, 24 and 48 h growth, relative to the planktonic conditions at each time point. Plus signs (+) indicate a gene’s benefit to biofilm fitness and minus signs (-) indicate its detrimental effect on biofilm fitness. (b) Pathways that affect biofilm fitness in *E. coli* or *S*. Typhimurium and those important in both species.

Analysis of the TraDIS-*Xpress* data found 78 genes implicated in biofilm formation in *S*. Typhimurium, compared to the 48 genes identified in *E. coli* (Fig. 1, Table S1, available in the online version of this article). The variation of sequence reads per insertion site between replicates was low for both the *E. coli* and *S*. Typhimurium TraDIS-*Xpress* datasets (Fig. S1), which indicates a high degree of reproducibility across all experimental conditions. As with our previous findings for *E. coli *, the pathways important for biofilm formation in *S*. Typhimurium changed over time as the biofilm develops. In *S*. Typhimurium biofilms after 12 h growth, genes involved in adhesion, fimbriae expression, cellulose biosynthesis and amino acid biosynthesis were beneficial to biofilm fitness. As the biofilm matured, pathways conferring protease activity, flagella biosynthesis, cAMP biosynthesis, respiration, purine biosynthesis, LPS biosynthesis, as well as various stress-response transcriptional regulators and transcription factors affected the fitness of biofilms (grown for 24 h). After 48 h growth, genes involved in biofilm matrix biosynthesis, fimbriae expression, flagella biosynthesis, respiration, transmembrane transport, LPS biosynthesis and ribosomal modification were beneficial to mature biofilm fitness in *S*. Typhimurium.

**Fig. 2.**
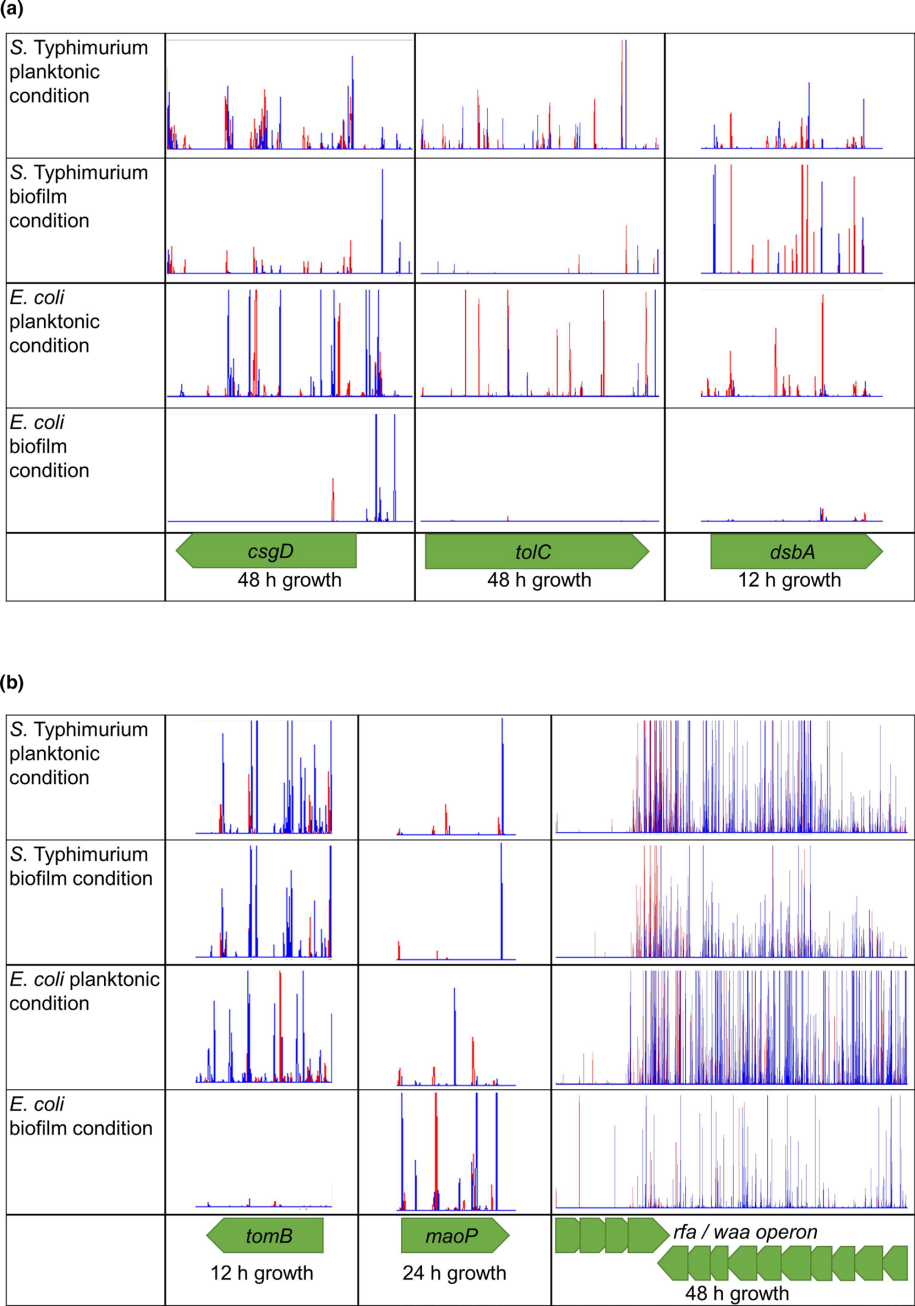
Transposon insertion sites and frequencies in planktonic and biofilm conditions plotted with BioTraDIS in Artemis. Red peaks show where the transposon-located promoter is facing left-to-right, and blue peaks show it facing right-to-left. For all plot files, the *Y*-axes have been normalized for each locus to show relative differences in insert abundance between conditions. Conditions with and without promoter induction have been combined and one of two independent replicates is shown. (a) Insertion sites in and around *csgD*, *tolC* and *dsbA* in planktonic and biofilm conditions in *E. coli* and *S*. Typhimurium. (b) Insertion sites in *tomB*, *maoP* and the *rfa* operon in planktonic and biofilm conditions in *E. coli* and *S*. Typhimurium.

**Fig. 3.**
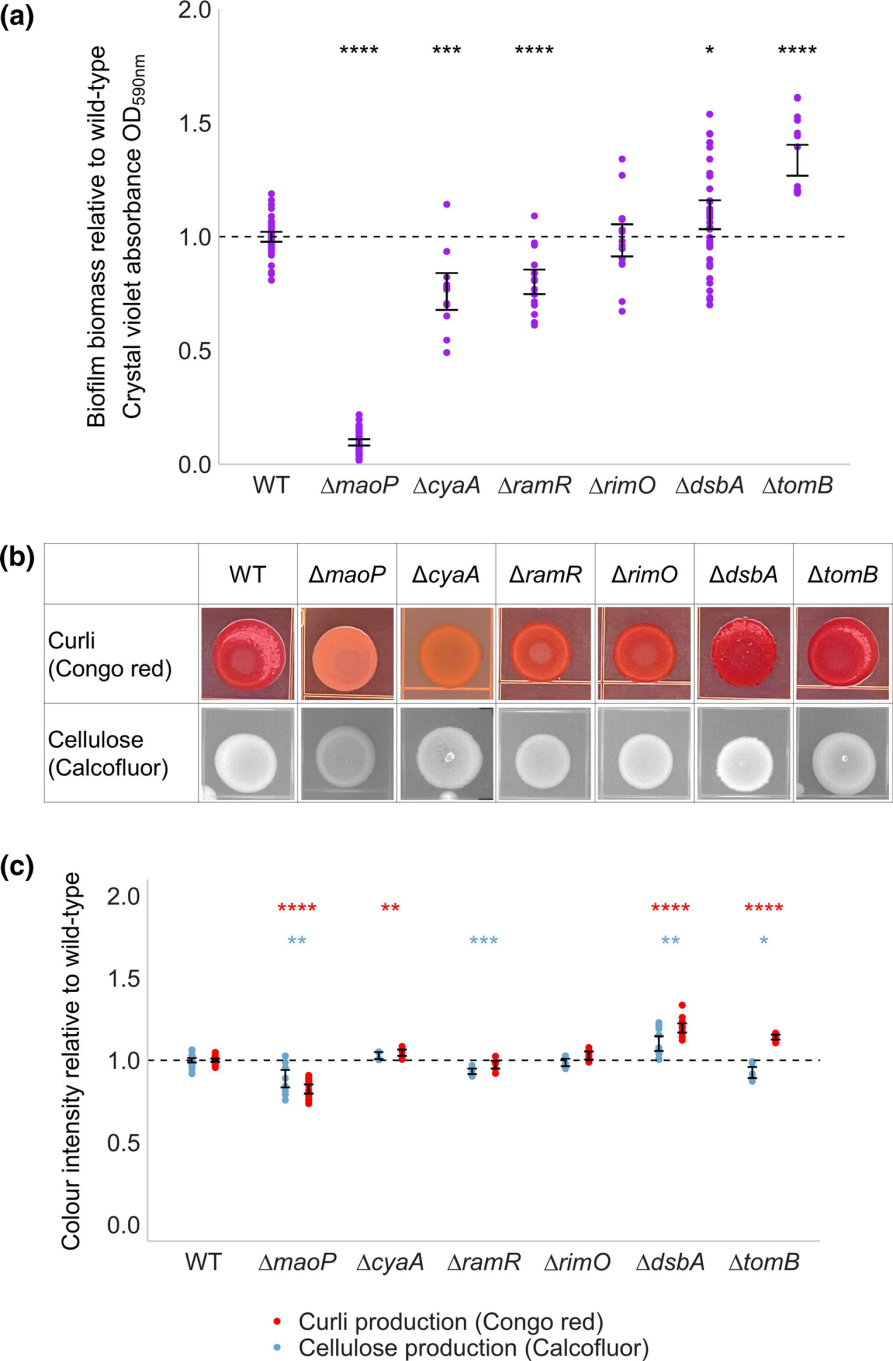
(a) Biofilm biomass of *S*. Typhimurium deletion mutants relative to the wild-type (WT). Points show relative biofilm biomass of a minimum of eight technical replicates across two biological replicates. (b) Curli and cellulose biosynthesis of *S*. Typhimurium deletion mutants measured by staining colonies with Congo red and calcofluor, respectively. Images are representative of two biological and two technical replicates. (c) Quantitative analysis of curli and cellulose production, measured from the colonies in (b). Points show relative colour intensity of colonies for six technical replicates across two biological replicates. For both graphs, error bars shows the 95 % confidence intervals. A significant difference in biofilm biomass to the WT is indicated by asterisks (Welch’s *t*-test, * = *P*<0.05; ** = *P*<0.01; *** = *P*<0.001; **** = *P*<0.0001).

Comparison of data between the species identified pathways important in biofilm formation in both, including type I fimbriae regulation, flagella biosynthesis, purine biosynthesis, curli production, LPS biosynthesis, sugar utilization, transmembrane transport and various similar transcriptional regulators (Fig. 1). Whilst there was a good overlap between pathways, only three individual genes were identified in both species at the same time points: these were *csgD*, *tolC* and *dsbA*. Of these, the genes encoding curli biosynthesis regulator CsgD and efflux channel TolC were beneficial to biofilm formation in both species after 48 h growth, however insertional inactivation of *dsbA*, encoding a disulphide oxidoreductase [8], affected the fitness in biofilms after 12 h growth in both species but in different ways (Fig. 2a). There were more insertions in *dsbA* in *S*. Typhimurium biofilm conditions at 12 h, and fewer insertions in *E. coli* biofilms at 12 and 24 h, relative to their planktonic conditions (Fig. 2a). In *E. coli *, *dsbA* had a positive effect on adhesion in the early biofilm and a detrimental effect on curli biosynthesis in the late biofilm. Deletion of *dsbA* in *S*. Typhimurium resulted in increased curli and cellulose biosynthesis compared to the wild-type (Fig. 3a, b and c).

Genes involved in LPS biosynthesis were important in both *S*. Typhimurium and *E. coli* biofilm formation. There were fewer insertions in the main LPS biosynthetic operons (*waa* in *E. coli* and *rfa* in *S*. Typhimurium) in biofilm conditions grown for 24 and 48 h, relative to planktonic controls (Fig. 2b). In *S*. Typhimurium, there were also more insertions upstream of *rfaJ* in biofilm conditions relative to planktonic after 12, 24 and 48 h growth, suggesting increased expression was beneficial for fitness throughout biofilm development. Increased expression of *rfaP* was also beneficial for fitness in biofilms grown for 24 h, and there were fewer insertions in this gene in the biofilm relative to the planktonic conditions after 48 h growth. However, not all genes involved in LPS biosynthesis were beneficial to the fitness of developing biofilms, as we found more insertions in *rfaG* after 24 and 48 h growth in *S*. Typhimurium biofilm conditions relative to the planktonic. We also found more insertions in *rfbJ*, involved in O-antigen biosynthesis, and *rfaL*, involved in ligation of the O-antigen to the LPS core, in biofilm conditions relative to planktonic after 24 and 48 h growth, respectively. Differences in the role of LPS in biofilm formation between *S*. Typhimurium and *E. coli* seen here may not reflect what would be seen in other *E. coli* strains due to the lack of O-antigen production in K-12 strains of *E. coli* [9].

Several transcription factors were found to affect biofilm formation in both *E. coli* and *S*. Typhimurium. In biofilms grown for 24 h, there were more insertions in *rpoS* in *S*. Typhimurium biofilms and more insertions in *dksA* in *E. coli* biofilms compared to planktonic conditions. Both genes encode transcription factors involved in stationary phase regulation, which are responsive to the stress signalling molecule ppGpp [10]. The Rcs phosphorelay, a multicomponent signalling system, was beneficial to biofilm fitness in both species. More in-frame insertions upstream of *rcsB* (resulting in overexpression) were observed in *S*. Typhimurium biofilms grown for 24 h and fewer mutants were observed within *rcsC* in *E. coli* biofilms after 48 h growth relative to planktonic culture. We found the homologous *mar* and *ram* global stress-response regulatory systems [11, 12] were beneficial for biofilm fitness at different points in biofilm formation in *S*. Typhimurium and *E. coli *. There were fewer mutants mapped to *ramA* in biofilms grown for 24 h and *ramR* in biofilms grown for 48 h compared to planktonic conditions in *S*. Typhimurium (*ramRA* is not present in *E. coli *), whereas *marR* was beneficial for biofilm fitness in *E. coli* after 12 h. Deletion of *ramR* in *S*. Typhimurium resulted in reduced biofilm biomass and cellulose biosynthesis after 48 h growth, consistent with the findings from the TraDIS-*Xpress* data (Fig. 3a, b and c).

### In *E. coli *, DNA housekeeping, cell division and motility regulation were more important to the fitness of the developing biofilm than in *S*. Typhimurium

Whilst many pathways were shared between the species in biofilm formation, there were also several differences between the two. TraDIS-*Xpress* identified genes involved in DNA housekeeping, cell division, c-di-GMP metabolism, motility regulation and antitoxin production were important in the fitness of *E. coli* but not *S*. Typhimurium when growing in a biofilm.

In *E. coli* biofilms grown for 24 h, DNA housekeeping genes *dam* and *maoP* were seen to have a significant effect on biofilm fitness, however no DNA housekeeping genes were predicted by the TraDIS-*Xpress* data to significantly impact biofilm formation by *S*. Typhimurium (Fig. 2b). We previously identified a novel role for *maoP* in biofilm formation, demonstrating its deletion resulted in a reduction in curli production and biofilm biomass in *E. coli* [5]. To further investigate the role of this gene on biofilm fitness in another member of the Enterobacteriaceae family, we disrupted the *maoP* homologue in *S*. Typhimurium and found it to have the same effect on biofilm formation in *S*. Typhimurium as in *E. coli *. In addition to a reduction in curli biosynthesis and biofilm biomass, deletion of *maoP* in *S*. Typhimurium also resulted in reduced cellulose biosynthesis, despite seeing no significant difference for this gene in the TraDIS-*Xpress* data (Fig. 3a, b and c). Antitoxin modulator *tomB* was found to benefit the fitness of *E. coli* biofilms grown for 12, 24 and 48 h, however this was not seen in *S*. Typhimurium from the TraDIS-*Xpress* data (Fig. 2b). In fact, contrary to the relationship between *tomB* and biofilm formation in *E. coli *, deletion of *tomB* in *S*. Typhimurium resulted in a significant increase in biofilm biomass and curli biosynthesis relative to the wild-type, but reduced cellulose biosynthesis (Fig. 3a, b and c).

In *S*. Typhimurium, genes involved in cellulose biosynthesis regulation, amino acid biosynthesis and ribosomal modification affected the fitness of the developing biofilm more than in *E. coli*

Pathways that were identified by TraDIS-*Xpress* to affect biofilm development in *S*. Typhimurium but not *E. coli* included cellulose biosynthesis regulation, cyclic AMP biosynthesis, amino acid biosynthesis and protease activity.

Increased expression of the adenylate cyclase *cyaA* [13] was beneficial to biofilm development, with more insertions seen upstream of this gene in biofilm conditions grown for 24 h relative to planktonic culture. In support of this, deletion of *cyaA* resulted in significantly reduced biofilm biomass relative to the wild-type (Fig. 3a). There was reduced curli biosynthesis in the Δ*cyaA* mutant as seen in Fig. 3b. This is not accurately represented in the quantitative data in Fig. 3c, which measured colony intensity and not the difference between orange and red colonies. cAMP is a secondary messenger molecule produced by *cyaA* that has been described to positively regulate *csgD* transcription [14], supporting our findings that cAMP biosynthesis is beneficial to biofilm fitness through regulation of matrix production.

Initial attachment and adhesion to surfaces is required for early biofilm formation. Insertions resulting in the overexpression of cellulose biosynthetic regulatory genes *gcpA* and *gcpG* [15] increased the fitness of S. Typhimurium biofilms after 12 h growth. Both genes encode GGDEF domain-containing proteins involved in c-di-GMP-dependent regulation of cellulose biosynthesis [15]. The importance of these genes so early in the biofilm life cycle implies a role for cellulose in attachment and adhesion to surfaces in *S*. Typhimurium.

Amino acid biosynthesis was important for early biofilm formation in *S*. Typhimurium, with fewer insertions in *hisC* [16] and *ilvH* [17] in biofilms grown for 12 h relative to planktonic conditions. IlvH has also been found to contain a potential c-di-GMP receptor [18], which is the secondary messenger molecule also involved in cellulose biosynthesis regulation. Another gene found to contain a c-di-GMP binding site was *rimO* [18], involved in ribosome methylthiolation [19]. TraDIS-*Xpress* identified more insertions upstream of *rimO* in biofilm conditions relative to planktonic after 48 h growth, suggesting that overexpression of *rimO* improved biofilm fitness. However, deletion of *rimO* resulted in no change in biofilm biomass or matrix production relative to the wild-type (Fig. 3a, b and c). These genes may affect different aspects of biofilm development not measured in these phenotypic assays, TraDIS-*Xpress* is an extremely sensitive tool to identify variations in fitness and further work may help identify how these genes impact biofilm development. The role of c-di-GMP in biofilm formation has been widely studied, and our results have identified c-di-GMP-regulated genes that benefit the fitness of the biofilm without directly affecting biomass or matrix production.

### Respiration is important for the fitness of the mature biofilm in *S*. Typhimurium

Genes involved in respiration were crucial for biofilm development in *S*. Typhimurium. Overexpression of *fumA*, part of the TCA cycle [20], was beneficial for biofilm fitness at 48 h. There were also fewer insertions in *pdhR* and *cra*, both involved in regulating the expression of genes in the TCA cycle and electron transport chain [21, 22]. Both *pdhR* and *cra* have also been associated with *csgD* promoter binding [23] and may negatively regulate curli biosynthesis.

**Fig. 4.**
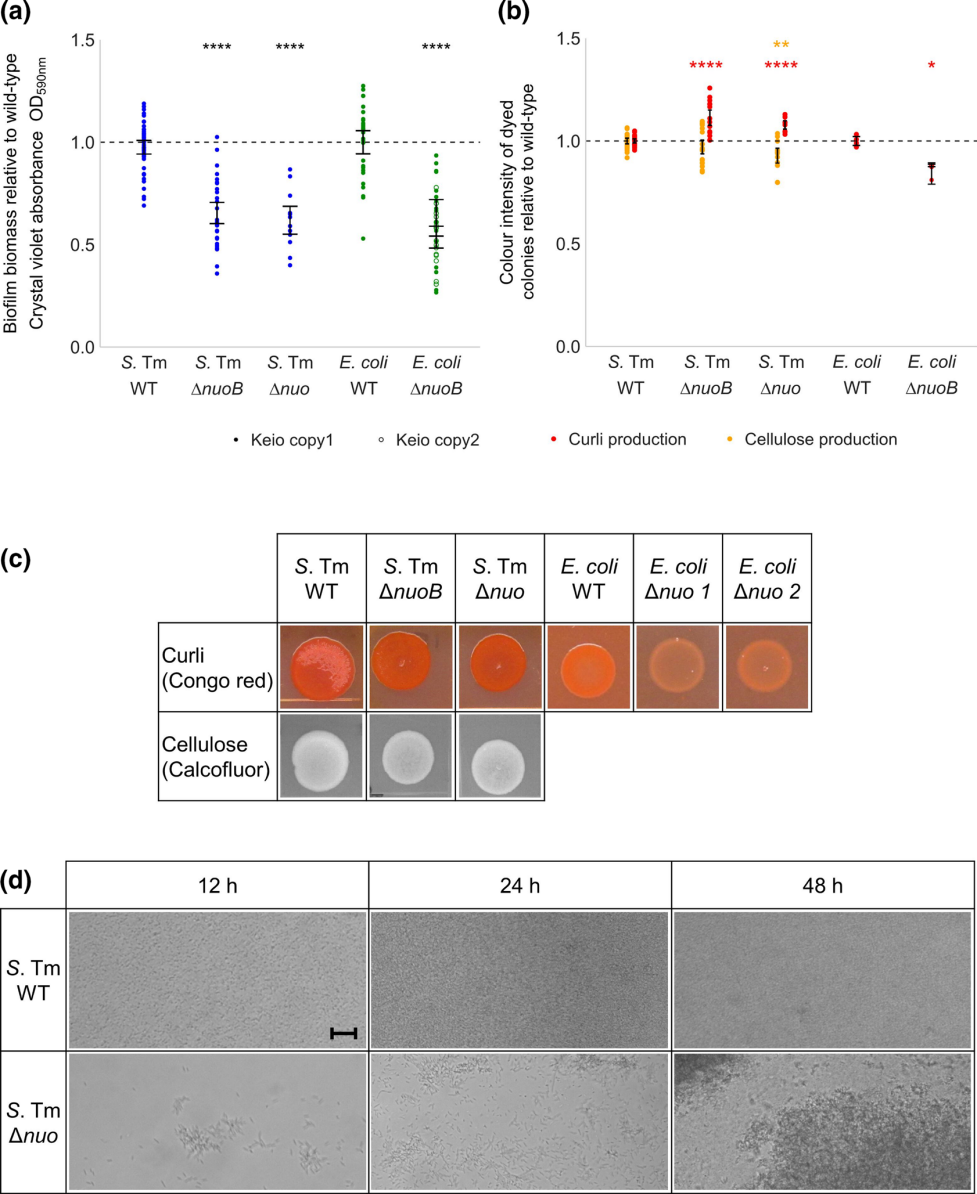
(a) Biofilm biomass of Δ*nuoB* and Δ*nuo* deletion mutants relative to wild-type (WT) S. Typhimurium and *E. coli *. (b) Curli and cellulose biosynthesis of wild-type, Δ*nuoB* and Δ*nuo* deletion mutants in *S*. Typhimurium and *E. coli *, measured from the images in (c). Images are representative of two biological and two technical replicates. For both graphs, points represent a minimum of four technical replicates across two biological replicates, and error bars show 95 % confidence intervals. A significant difference in biofilm biomass from the WT is indicated by asterisks (Welch’s *t*-test, * = *P*<0.05; ** = *P*<0.01; *** = *P*<0.001; **** = *P*<0.0001). (d) Biofilm formation of wild-type *S*. Typhimurium and the Δ*nuo* deletion mutant on glass analysed under flow conditions after 12, 24 and 48 h growth. Magnification, 20x. Scale bar indicates 10 µm.

We found 17 genes involved in the electron transport chain were important for the fitness of the growing and maturing *S*. Typhimurium biofilm after 24 and 48 h. The *nuo* operon, encoding the type I NADH dehydrogenase in the electron transport chain [24], contains 14 genes, ten of which we found to have fewer mutants in the biofilm conditions relative to planktonic culture after 24 or 48 h growth. In addition to this, there were also fewer insertions in *ygiN* and *menH*, involved in maintenance of the menaquinone pool [25, 26], in biofilm conditions after 24 h growth. A defined deletion of *nuoB* (Δ*nuoB,* near the beginning of the *nuo* operon) and a deletion of the majority of the *nuo* operon (Δ*nuo*, loss of *nuoB* to *nuoM*) both resulted in significantly reduced biofilm biomass and curli biosynthesis relative to wild-type *S*. Typhimurium, but no change in cellulose biosynthesis (Fig. 4a and c). Curli biosynthesis in the Δ*nuoB* and Δ*nuo* biofilms in *S*. Typhimurium is not accurately represented in the quantitative data in Fig. 4c, which measured colony intensity and not the difference between orange and red colonies. Cellulose biosynthesis was also significantly reduced compared to the wild-type but only in the Δ*nuo* mutant, not the Δ*nuoB* mutant (Fig. 4b). Analysis of *nuo*-deficient biofilms under flow conditions showed reduced adhesion after 12 and 24 h growth and reduced biofilm biomass relative to wild-type *S*. Typhimurium (Fig. 4d). The TraDIS-*Xpress* data did not detect a significant difference in insertions in the *nuo* operon between planktonic and biofilm conditions grown for 48 h in *E. coli *. However, deletion of *nuoB* in *E. coli* did reduce biofilm biomass and curli biosynthesis relative to the wild-type (Fig. 4a, b and c). This shows that respiration and the electron transport chain affect biofilm formation in both species, but the effect on the fitness of the biofilm appears to be more pronounced in *S*. Typhimurium.

### Genes not previously implicated in biofilm formation

TraDIS-*Xpress* identified 21 genes in *S*. Typhimurium that benefitted biofilm fitness that had not previously been linked to biofilm formation. These included *orfB*, *ybaN*, *yjiG*, STM14_0498, *STM14_1074, STM14_1157, STM14_1158, STM14_1440, STM14_1445, STM14_1764* and *STM14_3208* after 12 h growth, *tyrT*, *ybeF*, *ybeX* and *ygiN* after 24 h growth, and *ycgL*, *yliG*, *STM14_0653, STM14_2049, STM14_2856* and *STM14_3860* beneficial for biofilm formation after 48 h growth.

**Fig. 5.**
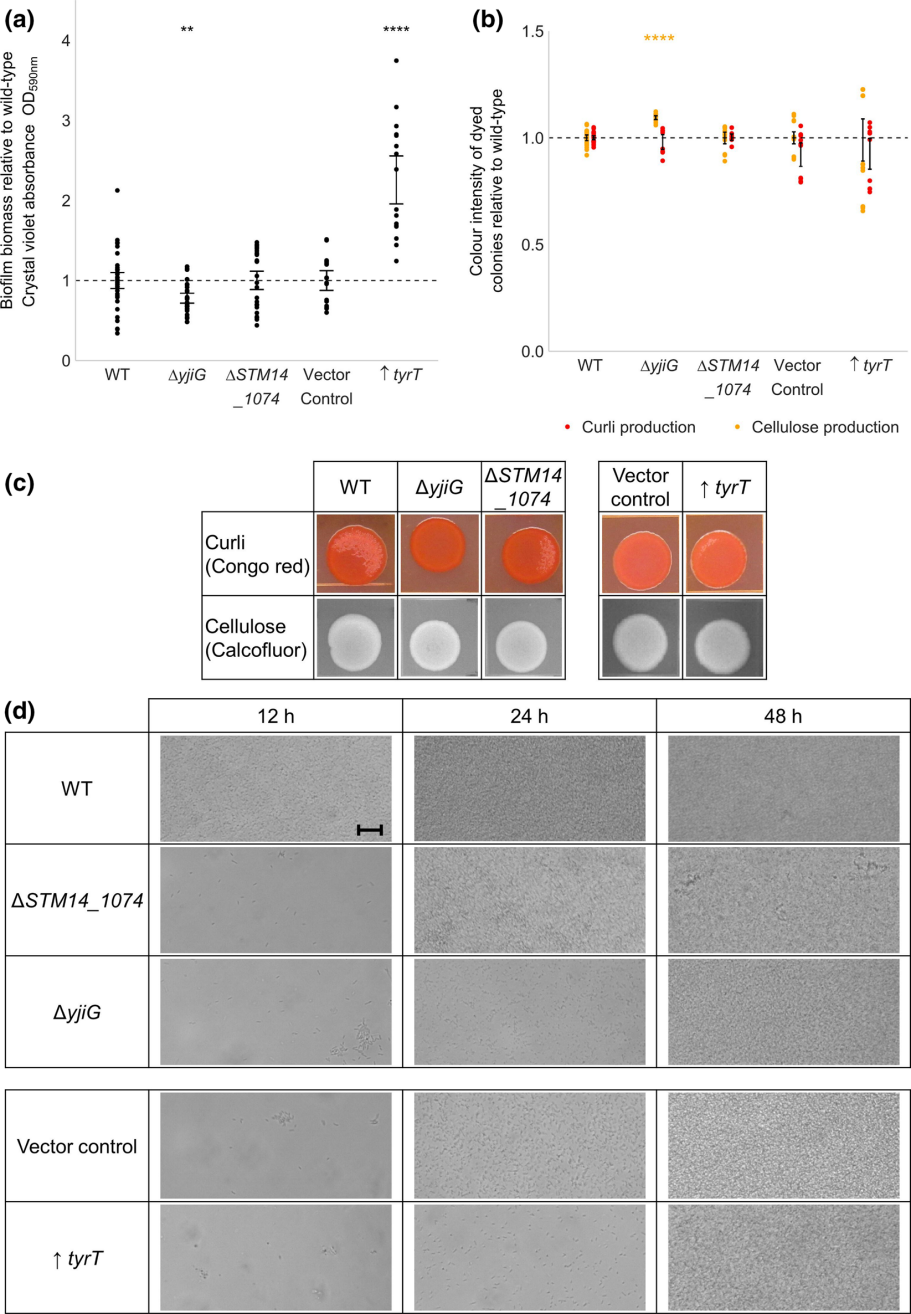
(a) Biofilm biomass of novel biofilm gene deletion and overexpression mutants in *S*. Typhimurium, relative to the wild-type (WT). (b) Curli and cellulose biosynthesis of mutants relative to the WT, measured from the images in (c). Images are representative of two biological and two technical replicates. For both graphs, points represent a minimum of four technical replicates across two biological replicates, and error bars show 95 % confidence intervals. A significant difference in biofilm biomass from the WT is indicated by asterisks (Welch’s *t*-test, * = *P*<0.05; ** = *P*<0.01; *** = *P*<0.001; **** = *P*<0.0001). (d) Biofilm formation of WT and deletion mutants on glass analysed under flow conditions after 12, 24 and 48 h growth. Magnification20x. Scale bar indicates 10 µm.

Of these, we chose to investigate three genes predicted from the data to have the largest effect on fitness in biofilm conditions in *S*. Typhimurium. These included the genes encoding putative transcriptional regulator STM14_1074 [27], inner membrane protein YjiG [28] and tyrosine tRNA TyrT [29]. Analysis of the TraDIS-*Xpress* data found fewer mutants in both *STM14_1074* and *yjiG* in biofilms grown for 12 h, relative to planktonic culture. This was supported by reduced biofilm biomass in a Δ*yjiG* mutant relative to wild-type S. Typhimurium (Fig. 5a) and reduced adhesion in both Δ*yjiG* and Δ*STM14_1074* mutants relative to the wild-type after 12 h growth under flow conditions (Fig. 5d). Deletion of Δ*yjiG* also resulted in increased cellulose biosynthesis relative to the wild-type (Fig. 5b and c), contrary to the TraDIS-*Xpress* data and other phenotypic measurements of biofilm formation, and therefore may affect many aspects of the developing biofilm. The TraDIS-*Xpress* data shows there were more transposon insertions upstream of *tyrT* in the biofilm conditions relative to the planktonic conditions, indicating that overexpression of *tyrT* was beneficial to biofilm fitness after 24 h growth. To investigate this, *tyrT* was inserted into the expression plasmid pBR322 under the IPTG-inducible *lac* operator (pBR322*lac*) in *S*. Typhimurium::*lacIZ* (LacI was chromosomally integrated into *S*. Typhimurium to control *lac* promoter activity with IPTG), and its effect on biofilm formation was examined relative to an empty vector control. Overexpression of *tyrT* (↑ *tyrT*) resulted in significantly increased biofilm biomass relative to the vector control (Fig. 5a), supporting our findings that expression of *tyrT* is beneficial for biofilm formation.

## Discussion

Temporal changes in gene expression are necessary for optimal fitness throughout the formation of a biofilm and we previously described various genes with temporal roles in biofilm formation in *E. coli *. Here we compared the genes and pathways required for biofilm development over time between *S*. Typhimurium and *E. coli *. Similar pathways identified to affect biofilm fitness in both species include type I fimbriae regulation, flagella biosynthesis, purine biosynthesis, curli production, LPS biosynthesis, sugar utilization, transmembrane transport and various similar transcriptional regulators.

We identified several differences in the genes and pathways required for optimal fitness of *E. coli* and *S*. Typhimurium biofilms. TraDIS-Xpress revealed the importance of *tomB* in biofilm formation in *E. coli *, where its deletion reduced biofilm fitness at all stages of biofilm development measured, through reducing biofilm biomass and curli biosynthesis. However in *S*. Typhimurium, deletion of *tomB* lead to increased biofilm biomass relative to the wild-type. TomB is an antitoxin to Hha, which interacts with H-NS to modulate its activity [30]. Deletion of *hns* has been reported to reduce curli biosynthesis through reduced *csg* transcription in *S*. Typhimurium [31], but led to an increase in *E. coli* [32]. It is possible that deletion of *tomB* removes inhibition of Hha, leading to increased H-NS activity and as a result impacts curli production and biofilm formation.

Pathways that affected biofilm fitness in *S*. Typhimurium but not *E. coli* included respiration, amino acid biosynthesis, cellulose biosynthesis regulation, cyclic AMP biosynthesis, iron acquisition and protease activity. This comparison between the two species revealed the relatively greater importance of genes involved in respiration for the fitness of the mature *S*. Typhimurium biofilm, where over a third of the genes identified to affect the fitness of *S*. Typhimurium biofilms grown for 48 h had a role in respiration. TraDIS-*Xpress* found 10 out of the 14 genes that make up the *nuo* operon, encoding the first NADH dehydrogenase of the electron transport chain, were beneficial for the fitness of the mature biofilm. Previous work found that the *nuo* operon was upregulated in *Acinetobacter baumannii* biofilms [33] and is essential for root colonization by *Pseudomonas fluorescens* [34], but we believe this study is the first to describe the effect of the *nuo* operon on biofilm matrix production in this way. Two other genes, *ygiN* and *menH,* were found to benefit biofilm formation after 24 h growth. These genes are involved in maintaining a pool of menaquinone, which is primarily used for electron transport in anaerobic conditions, and indicates anaerobic respiration is also important for optimal fitness in the biofilm.

Some of the differences in biofilm development between *E. coli* and *S*. Typhimurium identified here may be explained by known mutations in *E. coli* K-12 strains. An early stop codon in *bcsQ* prevents normal cellulose biosynthesis in *E. coli* K-12 strains [35], which makes up part of the biofilm matrix in other *E. coli* strains as well as in *S*. Typhimurium. The curli biosynthetic regulator CsgD also induces cellulose biosynthesis [36], which may explain why *csgD* and multiple genes that affect its expression (such as *dsbA* [37], *cyaA* [14]) were found to be more important at earlier time points in the development of *S*. Typhimurium biofilms relative to *E. coli* biofilms. Additionally, *E. coli* K-12 strains produce an incomplete LPS without an O-antigen [9]. TraDIS-*Xpress* found that genes involved in O-antigen synthesis and ligation were detrimental to the fitness of *S*. Typhimurium biofilms after 24 or 48 h growth. This supports previous work on O-antigen biosynthesis in *Salmonella,* which found that its disruption can be beneficial for biofilm biomass formation, as it can act as a surfactant that inhibits biofilm formation [38]. Other studies have however reported the opposite to be true, that O-antigen biosynthesis is beneficial for biofilm fitness and disruption of *galE* reduced biofilm formation on biotic surfaces, but this may be due to its role in the production of other exopolysaccharides rather than solely O-antigen biosynthesis [39, 40]. The relationship between O-antigen biosynthesis and biofilm formation is clearly complex and its relative importance is likely to vary between differing environmental conditions, strains and species.

We previously described how deletion of *maoP* reduced biofilm biomass and matrix biosynthesis in *E. coli *. Despite seeing no significant signal for this in the TraDIS-*Xpress* data in *S*. Typhimurium, we found a defined deletion mutant lacking *maoP* had the same affect in *S*. Typhimurium. We were the first to describe the effect of *maoP* on biofilm formation [5] and have not yet characterized the exact mechanism through which it affects biofilm formation in Enterobacteriaceae. It is possible the relative impact of *maoP* deletion varies between the species resulting in no strong difference in fitness in the *Salmonella* TraDIS-*Xpress* data. Further characterization of how *maoP* impacts development of the biofilm will be crucial to provide an explanation for this finding.

We identified 21 genes that had not previously been associated with biofilm formation in *S*. Typhimurium, and laboratory validation supported the prediction for roles of these genes in biofilm formation. Deletion of *STM14_1074* and *yjiG* reduced adhesion to glass under flow conditions after 12 h growth relative to the wild-type and overexpression of *tyrT* improved the fitness of biofilms grown for 24 h and resulted in a small increase in curli biosynthesis. Further characterization of these genes is warranted to determine the exact mechanism through which they affect biofilm development.

The model system used in this study compared planktonic and biofilm conditions from cells in the same well, allowing for a direct comparison of the mutants who could and could not form a biofilm. We expected to recover fewer mutants over time as they reach the limit of their biofilm-forming capabilities. However, we did not see any difference in the number of mutants recovered at each time point. Additionally, we were surprised to identify mutants at the later time points that were found to not be able to form a biofilm at the earlier time points. We think this can be explained in two ways; we will never capture all the mutants present in any population and so, whilst we can see a large reduction in mutants at a given position within the genome at a time point, there may be a small number remaining. If these have a big benefit later, they will expand and this will be visible in the data. Alternatively, the experiments (planktonic and biofilm) were grown in the same tubes – mutants with poor biofilm fitness at an early time point may be seeded from the planktonic pool later on, if they then have a benefit, they will be able to grow in the biofilm and we will then detect them again. Therefore biofilm conditions were compared to planktonic conditions at each time point, rather than comparing biofilm conditions to each other.

This comparison of gene essentiality and expression in the biofilm between *S*. Typhimurium and *E. coli* has revealed new information about biofilm fitness in both species, highlighting species-specific requirements as well as common pathways important in biofilm formation. This model system has successfully identified genes that affect biofilm fitness in two defined reference strains, paving the way for further investigations using wider sets of species and strains to allow broader assessments of ‘core’ and strain-specific genes. To the best of our knowledge, this work is the first to determine the role of the *nuo* operon in biofilm formation in *S*. Typhimurium and *E. coli *, as well as the first to identify a panel of 21 other genes not previous known to be involved in biofilm formation in *S*. Typhimurium. This work demonstrates the importance of comparing biofilm formation between different species to develop a deeper understanding of the core requirements for biofilm formation as well as species-specific differences.

## Methods

### Transposon mutant library in *S*. Typhimurium

The transposon used in previous TraDIS-*Xpress* experiments contains an outwards-facing *tac* promoter, the expression of which is controlled by IPTG and LacI in *E. coli *. However, *lacI* is not native to the *S*. Typhimurium genome, therefore *lacI* and the downstream reporter gene *lacZ* were integrated into the chromosome of *S*. Typhimurium strain 14 028*S* following the gene doctoring approach outlined by Holden *et al.* [41]. This strain was then grown to mid-logarithmic growth phase in 400 ml 2xYT broth supplemented with 0.7 mM EDTA. Bacteria were grown to an OD_600nm_ between 0.2 and 0.25 and were centrifuged at 4 °C for 10 min at 3000 ***g***. The supernatant was discarded, and pellets were washed three times with 10 % glycerol. Cells were resuspended in 600 µl 10 % glycerol and 60 µl aliquots were made in tubes on ice. These tubes contained 2 µl nuclease-free water, 2 µl TypeOne Restriction Inhibitor (Cambio) and 0.4 µl transposome. The transposome was made with 2 µl transposon DNA (described by Yasir *et al*. [42]) at a concentration of 100 ng µl^−1^, 2 µl 100 % glycerol and 4 µl EZ-Tn5 transposase (Epicentre). The transposome was electroporated into the cells using prechilled sterile 2 mm electrode gap cuvettes (Geneflow) and a Bio-Rad GenePusler II set to 2.4 kV and 200 Ω. Cells were immediately recovered in 1 ml SOC media (NEB). Following a 2 h recovery at 37 °C, five transformations were pooled and plated per bioassay plate (245×245 mm), containing LB agar supplemented with 50 µg ml^−1^ kanamycin. A 1 : 100 dilution of each pool was plated on a smaller plate to aid in calculating the size of the mutant library. Plates were incubated at 37 °C overnight. The following day, the number of colonies per plate was calculated and the bacteria were resuspended from the plate in LB broth. An equal volume of 100 % glycerol was added to this and 500 µL aliquots were made in cryotubes for storage at −20 °C.

### Biofilm model conditions

The model used to investigate biofilm formation over time in *S*. Typhimurium was identical to that used in our investigations with *E. coli* [5]. Parallel cultures of 5 ml LB broth (without salt) were inoculated with approximately 10^7^ cells from the pooled *S*. Typhimurium mutant library. This was done with and without 1 mM IPTG to induce expression from the transposon-located promoter. Cultures were grown in six-well plates with each well containing 40 sterile 5 mm glass beads (Sigma) per well and incubated at 30 ˚C with light agitation. After 12, 24 and 48 h post-inoculation, 1 ml of planktonic sample was collected from two wells and 70 beads were taken from two wells to constitute the biofilm sample. Planktonic and biofilm samples were taken from the same wells. Beads were washed twice in sterile PBS and vortexed in tubes containing PBS to resuspend cells from the biofilm. Both planktonic and biofilm samples were centrifuged at 2100 ***g*** to form pellets for DNA extraction. All conditions were run with two independent identical replicates.

### TraDIS-*Xpress* sequencing and informatics

DNA was extracted from pellets following the protocol described by Trampari *et al*. [43] and customized sequencing libraries were prepared in the same way as for the *E. coli* data. DNA was fragmented using the MuSeek DNA fragment library preparation kit (ThermoFisher) and were amplified by PCR using customized sequencing primers specific for the transposon tag. Fragments between 300 and 500 bp were size selected using AMPure beads (Beckman Coulter) and nucleotide sequences were generated using a NextSeq 500 and a NextSeq 500/550 High Output v2 kit (75 cycles) (Illumina). Between 2.7 and 22 million reads were obtained per condition.

### Informatics

Fastq files were aligned to the *S*. Typhimurium strain 14 028*S* reference genome (CP001363, modified to include chromosomally integrated *lacIZ*) using the BioTraDIS (version 1.4.3) software suite [44]. This programme creates plot files of insertion sites in the genome for planktonic and biofilm conditions over time. Significant differences (*P*<0.05, after correction for false discovery) in insertion frequencies per gene between planktonic and biofilm conditions at each time point were determined using the tradis_comparison.R command (part of the BioTraDIS toolkit) and AlbaTraDIS (version 1.0.1) [45]. Conditions with and without IPTG were combined for initial analysis and inserts predicted to only impact fitness of planktonic growth were excluded. For all candidate loci, plot files were examined manually in Artemis (version 17.0.1) [46] to validate the predictions of the automated analysis software.

### Validation experiments

We selected a series of genes for validation by creating defined deletion mutants to conform phenotypic impacts predicted by TraDIS-Xpress; these were selected to include the main pathways seen to differ between species. Single gene deletion mutants were made by gene doctoring [47] using plasmids designed by Thomson *et al*. [48] and confirmed by whole-genome sequencing. Overexpression constructs were made by restriction digestion and ligation of the gene of interest into pBR322 under the control of the *lac* promoter, which was incorporated into the primers used to amplify the gene of interest. This construct was confirmed by Sanger sequencing and transformed into a strain of *S*. Typhimurium, which expresses *lacI* to allow titratable control of the *lac* promoter through the addition of IPTG [41]. Mutants lacking or overexpressing genes of interest were assayed for their ability to form a biofilm relative to the wild-type. Biofilm biomass was investigated with crystal violet assays, where mutants were grown in 200 µl LB broth without salt for 48 h in polystyrene 96-well plates. The planktonic culture was removed, plates were rinsed and drained, and the resulting biomass was stained with 200 µl 0.1 % crystal violet for 10 min. The plates were rinsed with water to remove residual crystal violet, drained, and 200 µl 70 % ethanol was added to solubilize the stained biofilm. Absorbance was measured at 590 nm with a FLUOstar Omega plate reader (BMG Labtech). Biofilm matrix composition was determined by spotting 5 µl of bacterial culture (normalised to 10^7^ c.f.u. ml^−1^) on LB agar without salt supplemented with 40 µg ml^−1^ Congo red or 200 µg ml^−1^ Calcofluor to investigate curli or cellulose biosynthesis, respectively. For genes with unknown mechanisms by which they impact biofilm formation, we visualized biofilm development over time under flow conditions using a Bioflux system. LB broth without salt was fed into the flow cells at five dyne/cm^2^ and approximately 10^7^ cells were seeded into the chamber. The plate was incubated with no flow at room temperature for two and a half hours to allow attachment, and subsequently incubated at 30 °C at a flow rate of 0.3 dyne/cm^2^. Images of the growing biofilms were captured after 12, 24 and 48 h growth with a YenCam 3500 attached to an inverted light microscope. To compare growth kinetics between deletion mutants and the wild-type, overnight cultures were diluted to an optical density (OD_600nm_) of 0.1 in LB broth in a 96-well plate and OD_600nm_ was measured every 15 min for 24 h.

## Supplementary Data

Supplementary material 1
