# Supplementary material for: Comparison of the genetic basis of biofilm formation between Salmonella Typhimurium and Escherichia coli
Source: Microb Genom. 2022 Nov 3;8(11):mgen000885. doi: 10.1099/mgen.0.000885 (PMC9836088; doi:10.1099/mgen.0.000885)
Supplement: Supplementary material 1 [file mgen-8-885-s001.pdf]

Supplementary data

Table S1: Genes determined by TraDIS-*Xpress* to be important for biofilm formation in *E. coli* and *S.* Typhimurium (*S.* Tm)

Bacteria	Pathway	Gene	Time point	Difference in insertions in biofilm condition relative to planktonic condition		Significantly different phenotype from wild type?					Ref
				Log fold change *	Observed change	Biomass	Aggregation	Matrix production	Adhesion	Biofilm architecture	
**Both *E. coli* and *S.* Tm**	Curli biosynthesis	*csgD*	12h (*S.* Tm) 24h (*E. coli*), 48h (Both)	-1.4 (*E. coli*)	Increased expression beneficial at 12h and 48h in *S.* Tm, Increased expression beneficial at 24h in *E. coli*, Fewer insertions at 48h in *E. coli*	Reduced (in *E. coli*)	Reduced (in *E. coli*)	Reduced curli (in *E. coli*)			(Barnhart and Chapman, 2006)
	Protein modification	*dsbA*	12h (Both) 24h (*E. coli*)	-0.7 (*E. coli*) (*S.* 1.8 Tm) -3.0 (*E. coli*)	Fewer insertions in *E. coli*, More insertions in *S.* Tm	No change in *E. coli*, Increased biomass in *S.* Tm	Increased (in *E. coli*)	Increased curli in both, Increased cellulose in *S.* Tm			(Bardwell, 1994)
	Transmembrane transport	*tolC*	48h	-2.9 (*E. coli*) -0.7 (*S.* Tm)	Fewer insertions in both	No change (in *E. coli*) Reduced (in *S.* Tm)	Reduced (in *E. coli*)	No change in curli (*E. coli*) Reduced curli (*S.* Tm)			(Morona et al., 1983)

E. coli	Cell division	*zapE*	48h	-3.4	Fewer insertions	No change	Increased	No change in curli	Reduced	No change	(Marteyn et al., 2014)
	c-di-GMP metabolism	*rcdA*	48h	-0.8	Fewer insertions	Reduced		Reduced curli			(Pfiffer et al., 2019)
		pdeF	48h	-0.3	Fewer insertions						(Lacey et al., 2010)
	Curli biosynthesis	*csgC*	24h, 48h	-1.6 -0.6	Fewer insertions						(Barnhart and Chapman, 2006)
		csgE	12h, 48h	-2.5 -1.6	Fewer insertions	Reduced		Reduced curli			
		csgF	48h	-4.8	Fewer insertions						
	DNA housekeeping	*dam*	24h	-3.9	Fewer insertions	No change	Reduced	No change in curli			(Szyf et al., 1984)
		maoP	24h	1.6	More insertions	Reduced (in both *E. coli* and *S.* Tm)	Reduced	Reduced curli in *E. coli*, Reduced curli and cellulose in *S.* Tm	Reduced	Reduced density and biomass	(Valens et al., 2016)
	Flagella-associated motility	*flhD*	24h, 48h	-3.9 -2.6	Increased expression beneficial	No change	No change	No change in curli			(Fitzgerald et al., 2014)
		flhC	48h	-4.1	Fewer insertions	No change	Reduced	No change in curli			
		flgD	24h	-3.0	Fewer insertions	No change	No change	No change in curli			(Macnab, 1992)
		fliE	48h	-4.7	Fewer insertions	No change	Reduced	No change in curli			
		hdfR	12h, 24h	3.8 2.4	More insertions	Reduced	No change	Reduced curli			(Ko and Park, 2000)
		lrhA	12h, 24h, 48h	2.0 3.2 2.3	More insertions	No change	Reduced	No change in curli	Increased	Early microcolony formation, reduced in	(Lehnen et al., 2002)

										the mature biofilm	
	LPS	*wzzB*	48h	-1.4	Fewer insertions	Reduced		No change in curli			(Stenberg et al., 2005)
	Oxidised protein repair	*msrQ*	48h	-0.4	Fewer insertions	No change		No change in curli			(Gennaris et al., 2015)
	Purine ribonucleotide biosynthesis	*purD*	48h	-4.3	Fewer insertions	Reduced	No change	Reduced curli	No change	Reduced microcolony formation	(Zhang et al., 2008)
		purE	48h	-5.7	Fewer insertions	Reduced	Increased	Reduced curli			
		purH	48h	-3.2	Fewer insertions						
		purL	48h	-3.1	Fewer insertions						
	rRNA methyltransferase	*rlmI*	12h	-3.8	Fewer insertions	Reduced	No change	No change in curli			(Herzberg et al., 2006)
	RNase III regulator	*ymdB*	24h, 48h	-0.5 -2.5	Fewer insertions	Reduced	Increased	No change in curli			(Kim et al., 2013)
	Sugar metabolism and transport	*sgbE*	48h	-2.5	Fewer insertions	No change		No change in curli			(Yew and Gerlt, 2002)
	Toxin-antitoxin system	*tomB*	12h, 24h, 48h	-0.5 -0.4 -1.6	Fewer insertions	Reduced in *E. coli*, Increased in *S*. Tm	Reduced	Reduced curli in *E. coli*, Increased curli in *S*. Tm, Reduced cellulose in *S*. Tm	Increased	Early microcolony formation, reduced in the mature biofilm	(Garcia-Contreras et al., 2008)
	Transcriptional regulators and signalling systems	*dksA*	12h, 24h	4.4 2.9	More insertions	Reduced	Reduced	Reduced curli	Increased	Reduced microcolony formation	(Girard et al., 2018, Lemke et al., 2009, Mallik et al., 2006)

		leuO	12h, 48h	-0.6	Increased expression beneficial at 12h, Fewer insertions at 48h	Reduced	Reduced	No change in curli	No change	Reduced microcolony formation	(Dillon et al., 2012)
		marR	12h	-4.1	Fewer insertions	Reduced	No change	No change in curli			(Alekshun and Levy, 1999)
		ompR	24h, 48h	-0.8 -4.7	Fewer insertions	Reduced	Reduced	Reduced curli			(Cai and Inouye, 2002)
		lrp	48h	-5.9	Fewer insertions	Reduced	Reduced	Reduced curli			(Calvo and Matthews, 1994)
		gadW	48h	-1.1	Fewer insertions	No change	No change	No change in curli			(Tucker et al., 2003)
		rcsC	48h	-2.9	Fewer insertions	Reduced		No change in curli			(Ferrières and Clarke, 2003)
	Transmembrane transport, porins and channels	*mscL*	48h	-0.9	Fewer insertions						(Sukharev et al., 1994)
		ompF	48h	-2.7	Fewer insertions	Reduced		No change in curli			(Cai and Inouye, 2002)
		fadL	48h	-1.5	Fewer insertions						(Nunn and Simons, 1978)
	tRNA modification	*truA*	24h, 48h	-3.3 -5.9	Fewer insertions	No change	Increased	No change in curli	No change	Increased filamentation after 24- and 48-hours growth	(Hamma and Ferré-D'Amaré, 2006)
	Type 1 fimbriae	*fimB*	12h, 24, 48h	-0.4 -1.3 -2.1	Fewer insertions and increased expression beneficial	No change	Reduced	No change in curli			(Klemm, 1986)

					at all time points						
		fimE	12h, 24, 48h	1.5 3.3 2.6	More insertions	Reduced	Reduced	No change in curli			
		fimC	48h	-1.3	Fewer insertions						(Allen et al., 2012)
		fimD	24h, 48h	-2.3 -2.1	Fewer insertions						
	Putative fimbrial-like protein	*ydeR*	48h	-2.4	Fewer insertions						(Da Re et al., 2013)
	Unknown	*yigZ*	12h	-2.8	Fewer insertions	No change	Increased	No change in curli	No change	No change	(Park et al., 2004)
		yebB	48h	-2.3	Fewer insertions						(Schurr et al., 1993, Alper and Stephanopoulos, 2008)
		yedN	48h	-1.7	Fewer insertions						(Goodall et al., 2018)
		yjbL	48h	-2.8	Fewer insertions	Reduced		No change in curli			(Herzberg et al., 2006)
		ykgJ	12h		Reduced expression beneficial (increased expression of antisense mRNA beneficial)	No change	Increased	No change in curli	No change	Increased filamentation after 24- and 48-hours growth	(Kacharia et al., 2017)
**S. Typhimurium (*S.* Tm)**	Adhesin	*sadA*	12h		Increased expression beneficial						(Raghunathan et al., 2011)
	Amino acid synthesis	*ilvH*	12h	-2.5	Fewer insertions						(Squires et al., 1981)

		hisC	12h	-1.6	Fewer insertions						(Schembri et al., 2003)
	Biofilm matrix component	*bapA*	48h		Increased expression beneficial						(Latasa et al., 2005)
	Polysaccharide biosynthesis	*gcpA*	12h		Increased expression beneficial						(Garcia et al., 2004)
		gcpG	12h		Increased expression beneficial						
		manA	48h	-2.4	Fewer insertions						(Kwan et al., 2018, Li et al., 2017)
	Secondary messenger molecular metabolism	*cyaA*	24h		Increased expression beneficial	Reduced		Reduced curli			(Roy and Danchin, 1982)
	Curli biosynthesis	*csgB*	48h		Increased expression beneficial						(Barnhart and Chapman, 2006)
	Ethanolamine utilisation	*eutQ*	12h, 24h, 48h		Reduced expression beneficial (increased expression of antisense mRNA beneficial)						(Moore and Escalante-Semerena, 2016)
	Fimbriae	*fimY*	12h, 48h		Increased expression beneficial						(Saini et al., 2009)
		fimW	48h	1.8	More insertions						

		fimZ	48h		Increased expression beneficial							
		fimA	12h, 48h		Increased expression beneficial							(Allen et al., 2012)
	Flagella-associated motility	*flgA*	48h	-1.8	Fewer insertions							(Macnab, 1992)
		flgB	24h	-9.8	Fewer insertions							
		flgF	48h	-0.9	Fewer insertions							
		flgN	48h	-1.8	Fewer insertions							
	Iron acquisition	*ybaN*	12h	-8.6	Fewer insertions							(Seo et al., 2014)
	LPS	*rfaJ*	12h, 24h, 48h		Increased expression beneficial							(Wang et al., 2015)
		rfaP	24h, 48h	-1.0	Increased expression beneficial at 24h, Fewer insertions at 48h							
		rfaG	24h, 48h	2.2 2.2	More insertions							
		rfaL	48h	0.8	More insertions							
		rfbJ	24h	2.5	More insertions							
	Outer membrane integrity	*yciB*	48h	-4.3	Fewer insertions							(Niba et al., 2008)
	Prosthetic group biosynthesis	*citG*	12h	-2.3	Fewer insertions							(Hynes and Murray, 2010)

	Protease	*clpS*	24h	-3.6	Fewer insertions						(Yeom et al., 2018)
	Purine ribonucleotide metabolism	*purK*	24h		Increased expression beneficial						(Zhang et al., 2008)
		nagD	48h	-3.6	Fewer insertions						(Tremblay et al., 2006)
	Quorum sensing	*STM14_2049/ cat*	48h		Increased expression beneficial						(Liao et al., 2019)
	Respiration	*nuoB*	24h,<br>48h	-1.0<br>-5.8	Fewer insertions	Reduced in both		Reduced curli in both	Reduced	Reduced density and biomass	(Archer and Elliott, 1995)
		nuoC	24h,<br>48h	-2.6<br>-0.8	Fewer insertions			Reduced curli and cellulose in a *nuo* operon deletion			
		nuoF	48h	-4.4	Fewer insertions						
		nuoG	24h,<br>48h	-0.8<br>-2.0	Fewer insertions						
		nuoH	48h	-9.6	Fewer insertions						
		nuoI	48h	-8.8	Fewer insertions						
		nuoJ	24h,<br>48h	-2.5<br>-1.9	Fewer insertions						
		nuoK	24h,<br>48h	-9.0<br>-1.9	Fewer insertions						
		nuoL	24h,<br>48h	-1.0<br>-1.1	Fewer insertions						
		nuoM	24h,<br>48h	-1.8<br>-2.8	Fewer insertions						
		menH.	24h	-2.2	Fewer insertions						(Meganathan and Kwon, 2009)

		fumA	48h		Increased expression beneficial						(Guest and Roberts, 1983)
		pdhR	48h	-2.7	Fewer insertions						(Ogasawara et al., 2007)
		cra	48h	-7.2	Fewer insertions						(Saier and Ramseier, 1996)
		ygiN	24h	-2.1	Fewer insertions						(Adams and Jia, 2005)
	Sugar import & degradation	*ulaC*	48h	-3.1	Fewer insertions						(Wu et al., 2016)
	Transcription factors	*rpoS*	24h	2.4	More insertions						(Gentry et al., 1993)
		ramA	24h	-4.0	Fewer insertions						(George et al., 1995)
		ramR.	48h	-3.7	Fewer insertions	Reduced		Reduced cellulose			(Abouzeed et al., 2008)
		rcsB	24h		Increased expression beneficial						(Majdalani and Gottesman, 2005)
		ybeF	24h		Increased expression beneficial						
		yfaX	12h	-1.4	Fewer insertions						
		STM14_1074	12h	-5.6	Fewer insertions	No change		No change	Reduced	No change from WT	(Qin et al., 2016)
	Transmembrane transport, porins and channels	*araJ*	12h	-2.0	Fewer insertions						(Reeder and Schleif, 1991)
		cysW	48h	-2.2	Fewer insertions						(Sirko et al., 1995)
		corC	24h	-2.3	Fewer insertions						(Gibson et al., 1991)

		ydeD	24h		Increased expression beneficial						(Dassler et al., 2000)
	Translation	*rimO*	48h		Increased expression beneficial	No change		No change			(Anton et al., 2008)
		tyrT	24h		Increased expression beneficial	Increased when overexpressed		No change when overexpressed	No change	No change from vector control	(Winston et al., 1979)
	Type 4 pili	*ppdC*	48h	-2.2	Fewer insertions						(Cisneros et al., 2012)
	Unknown	*orfB*	12h	-1.7	Fewer insertions						
		ycgL	48h	-2.2	Fewer insertions						
		ygbA	48h	-2.1	Fewer insertions						
		yjiG	12h	-6.2	Fewer insertions	Reduced		No change in curli, Reduced cellulose	Reduced	No change from WT	(Tang and Saier, 2014)
		STM14_0498	12h	-7.9	Fewer insertions						
		STM14_0634	12h		Increased expression beneficial						
		STM14_0653	48h	-2.5	Fewer insertions						
		STM14_1157	12h		Increased expression beneficial						
		STM14_1158	12h		Increased expression beneficial						

		STM14_1440	12h		Increased expression beneficial						
		STM14_1445	12h		Increased expression beneficial						
		STM14_1764	12h	-10.0	Fewer insertions						
		STM14_2856	48h	-0.8	Fewer insertions						
		STM14_3208	12h		Increased expression beneficial						
		STM14_3860	48h		Increased expression beneficial						
		STM14_5332	48h	-3.9	Fewer insertions						

* Log fold change is only shown for genes where there are differences in insertion frequency inside the coding region. Where the plot files generated by BioTraDIS show a difference in insertion frequency between the biofilm and planktonic conditions upstream or downstream of a gene, log fold change cannot easily by quantified and therefore the effect has been described in the column titled ‘observed change’. Significant differences in insertion frequencies have been manually verified with the plot files generated by TraDIS-*Xpress*.

Figure S1

a)

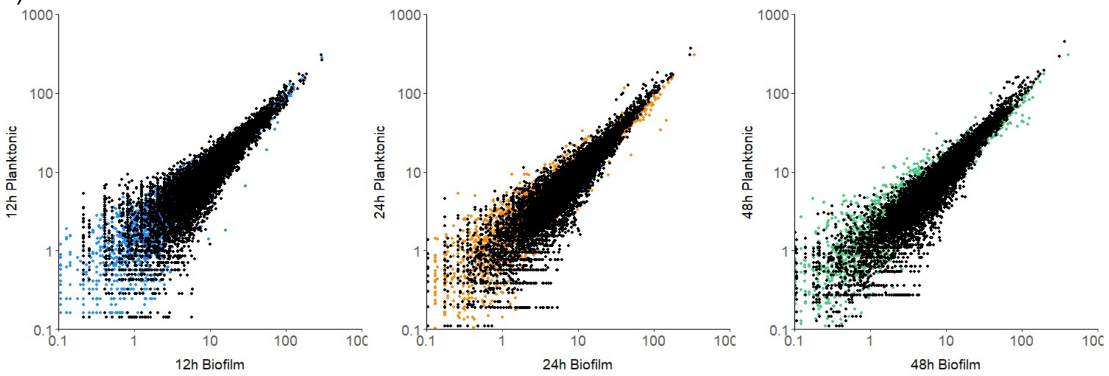

b)

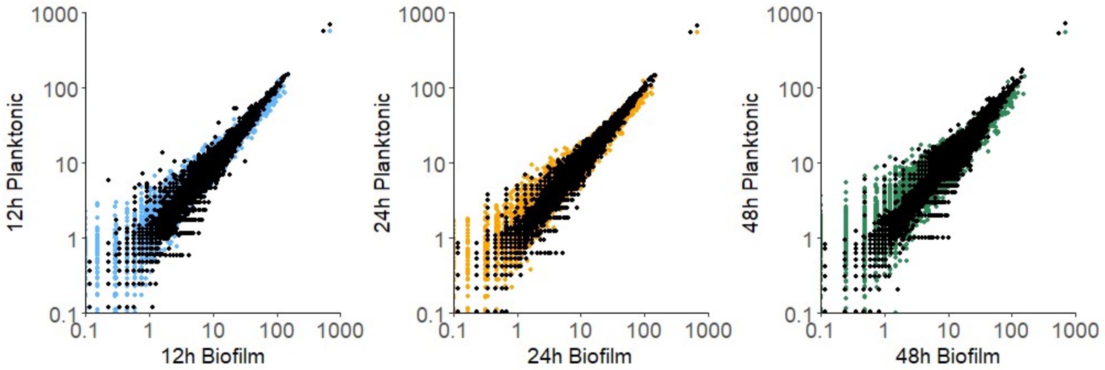

Figure S1: Insertion frequency per gene for the a) *E. coli* and b) *S*. Typhimurium transposon mutant library cultured under biofilm conditions (x-axis) compared to planktonic conditions (y-axis) for each time point (12, 24 and 48 hours). Black points represent the insertion frequency per gene for each replicate to show variation between replicates, and coloured points show the insertion frequency per gene of the biofilm condition versus the planktonic condition.

References for Supplementary data

ABOUZEED, Y. M., BAUCHERON, S. & CLOECKAERT, A. 2008. *ramR* Mutations Involved in Efflux-Mediated Multidrug Resistance in *Salmonella enterica* Serovar Typhimurium. *Antimicrobial Agents and Chemotherapy,* 52**,** 2428-2434.

ADAMS, M. A. & JIA, Z. 2005. Structural and biochemical evidence for an enzymatic quinone redox cycle in *Escherichia coli*: identification of a novel quinol monooxygenase. *J Biol Chem,* 280**,** 8358-63.

ALEKSHUN, M. N. & LEVY, S. B. 1999. Alteration of the Repressor Activity of MarR, the Negative Regulator of the *Escherichia coli marRAB* Locus, by Multiple Chemicals In Vitro. *Journal of Bacteriology,* 181**,** 4669-4672.

ALLEN, W. J., PHAN, G. & WAKSMAN, G. 2012. Pilus biogenesis at the outer membrane of Gram-negative bacterial pathogens. *Current Opinion in Structural Biology,* 22**,** 500-506.

ALPER, H. & STEPHANOPOULOS, G. 2008. Uncovering the gene knockout landscape for improved lycopene production in *E. coli*. *Applied Microbiology and Biotechnology,* 78**,** 801-810.

ANTON, B. P., SALEH, L., BENNER, J. S., RALEIGH, E. A., KASIF, S. & ROBERTS, R. J. 2008. RimO, a MiaB-like enzyme, methylthiolates the universally conserved Asp88 residue of ribosomal protein S12 in *Escherichia coli*. *Proc Natl Acad Sci U S A,* 105**,** 1826-31.

ARCHER, C. D. & ELLIOTT, T. 1995. Transcriptional control of the nuo operon which encodes the energy-conserving NADH dehydrogenase of *Salmonella* typhimurium. *Journal of bacteriology,* 177**,** 2335-2342.

BARDWELL, J. C. 1994. Building bridges: disulphide bond formation in the cell. *Mol Microbiol,* 14, 199-205.
BARNHART, M. M. & CHAPMAN, M. R. 2006. Curli Biogenesis and Function. *Annual Review of Microbiology,* 60, 131-147.
CAI, S. J. & INOUYE, M. 2002. EnvZ-OmpR interaction and osmoregulation in *Escherichia coli*. *J Biol Chem,* 277, 24155-61.
CALVO, J. M. & MATTHEWS, R. G. 1994. The leucine-responsive regulatory protein, a global regulator of metabolism in *Escherichia coli*. *Microbiol Rev,* 58, 466-90.
CISNEROS, D. A., PEHAU-ARNAUDET, G. & FRANCETIC, O. 2012. Heterologous assembly of type IV pili by a type II secretion system reveals the role of minor pilins in assembly initiation. *Molecular Microbiology,* 86, 805-818.
DA RE, S., VALLE, J., CHARBONNEL, N., BELOIN, C., LATOUR-LAMBERT, P., FAURE, P., TURLIN, E., LE BOUGUENEC, C., RENAULD-MONGENIE, G., FORESTIER, C. & GHIGO, J. M. 2013. Identification of commensal *Escherichia coli* genes involved in biofilm resistance to pathogen colonization. *PLoS One,* 8, e61628.
DASSLER, T., MAIER, T., WINTERHALTER, C. & BÖCK, A. 2000. Identification of a major facilitator protein from *Escherichia coli* involved in efflux of metabolites of the cysteine pathway. *Mol Microbiol,* 36, 1101-12.
DILLON, S. C., ESPINOSA, E., HOKAMP, K., USSERY, D. W., CASADESUS, J. & DORMAN, C. J. 2012. LeuO is a global regulator of gene expression in *Salmonella enterica* serovar Typhimurium. *Mol Microbiol,* 85, 1072-89.
FERRIÈRES, L. & CLARKE, D. J. 2003. The RcsC sensor kinase is required for normal biofilm formation in *Escherichia coli* K-12 and controls the expression of a regulon in response to growth on a solid surface. *Molecular Microbiology,* 50, 1665-1682.
FITZGERALD, D. M., BONOCORA, R. P. & WADE, J. T. 2014. Comprehensive Mapping of the *Escherichia coli* Flagellar Regulatory Network. *PLOS Genetics,* 10, e1004649.
GARCIA-CONTRERAS, R., ZHANG, X. S., KIM, Y. & WOOD, T. K. 2008. Protein translation and cell death: the role of rare tRNAs in biofilm formation and in activating dormant phage killer genes. *PLoS One,* 3, e2394.
GARCIA, B., LATASA, C., SOLANO, C., GARCIA-DEL PORTILLO, F., GAMAZO, C. & LASA, I. 2004. Role of the GGDEF protein family in *Salmonella* cellulose biosynthesis and biofilm formation. *Mol Microbiol,* 54, 264-77.
GENNARIS, A., EZRATY, B., HENRY, C., AGREBI, R., VERGNES, A., OHEIX, E., BOS, J., LEVERRIER, P., ESPINOSA, L., SZEWCZYK, J., VERTOMMEN, D., IRANZO, O., COLLET, J.-F. & BARRAS, F. 2015. Repairing oxidized proteins in the bacterial envelope using respiratory chain electrons. *Nature,* 528, 409-412.
GENTRY, D. R., HERNANDEZ, V. J., NGUYEN, L. H., JENSEN, D. B. & CASHEL, M. 1993. Synthesis of the stationary-phase sigma factor σs is positively regulated by ppGpp. *J Bacteriol,* 175, 7982-9.
GEORGE, A. M., HALL, R. M. & STOKES, H. W. 1995. Multidrug resistance in *Klebsiella pneumoniae*: a novel gene, *ramA*, confers a multidrug resistance phenotype in *Escherichia coli*. *Microbiology,* 141, 1909-1920.
GIBSON, M. M., BAGGA, D. A., MILLER, C. G. & MAGUIRE, M. E. 1991. Magnesium transport in *Salmonella* typhimurium: the influence of new mutations conferring Co2+ resistance on the CorA Mg2+ transport system. *Molecular Microbiology,* 5, 2753-2762.
GIRARD, M. E., GOPALKRISHNAN, S., GRACE, E. D., HALLIDAY, J. A., GOURSE, R. L. & HERMAN, C. 2018. DksA and ppGpp Regulate the σS Stress Response by Activating Promoters for the Small RNA DsrA and the Anti-Adapter Protein IraP. *Journal of Bacteriology,* 200, e00463-17.
GOODALL, E. C. A., ROBINSON, A., JOHNSTON, I. G., JABBARI, S., TURNER, K. A., CUNNINGHAM, A. F., LUND, P. A., COLE, J. A. & HENDERSON, I. R. 2018. The Essential Genome of *Escherichia coli* K-12. *mBio,* 9, e02096-17.

GUEST, J. R. & ROBERTS, R. E. 1983. Cloning, mapping, and expression of the fumarase gene of *Escherichia coli* K-12. *J Bacteriol,* 153**,** 588-96.
HAMMA, T. & FERRÉ-D'AMARÉ, A. R. 2006. Pseudouridine Synthases. *Chemistry & Biology,* 13**,** 1125-1135.
HERZBERG, M., KAYE, I. K., PETI, W. & WOOD, T. K. 2006. YdgG (TqsA) controls biofilm formation in *Escherichia coli* K-12 through autoinducer 2 transport. *J Bacteriol,* 188**,** 587-98.
HYNES, M. J. & MURRAY, S. L. 2010. ATP-Citrate Lyase Is Required for Production of Cytosolic Acetyl Coenzyme A and Development in *Aspergillus nidulans*. *Eukaryotic Cell,* 9**,** 1039-1048.
KACHARIA, F. R., MILLAR, J. A. & RAGHAVAN, R. 2017. Emergence of New sRNAs in Enteric Bacteria is Associated with Low Expression and Rapid Evolution. *Journal of Molecular Evolution,* 84**,** 204-213.
KIM, T., LEE, J. & KIM, K.-S. 2013. *Escherichia coli* YmdB regulates biofilm formation independently of its role as an RNase III modulator. *BMC microbiology,* 13**,** 266-266.
KLEMM, P. 1986. Two regulatory *fim* genes, *fimB* and *fimE*, control the phase variation of type 1 fimbriae in *Escherichia coli*. *The EMBO Journal,* 5**,** 1389-1393.
KO, M. & PARK, C. 2000. H-NS-Dependent regulation of flagellar synthesis is mediated by a LysR family protein. *J Bacteriol,* 182**,** 4670-2.
KWAN, G., PLAGENZ, B., COWLES, K., PISITHKUL, T., AMADOR-NOGUEZ, D. & BARAK, J. D. 2018. Few Differences in Metabolic Network Use Found Between *Salmonella enterica* Colonization of Plants and Typhoidal Mice. *Frontiers in microbiology,* 9**,** 695-695.
LACEY, M. M., PARTRIDGE, J. D. & GREEN, J. 2010. *Escherichia coli* K-12 YfgF is an anaerobic cyclic di-GMP phosphodiesterase with roles in cell surface remodelling and the oxidative stress response. *Microbiology,* 156**,** 2873-2886.
LATASA, C., ROUX, A., TOLEDO-ARANA, A., GHIGO, J. M., GAMAZO, C., PENADES, J. R. & LASA, I. 2005. BapA, a large secreted protein required for biofilm formation and host colonization of *Salmonella enterica* serovar Enteritidis. *Mol Microbiol,* 58**,** 1322-39.
LEHNEN, D., BLUMER, C., POLEN, T., WACKWITZ, B., WENDISCH, V. F. & UNDEN, G. 2002. LrhA as a new transcriptional key regulator of flagella, motility and chemotaxis genes in *Escherichia coli*. *Molecular Microbiology,* 45**,** 521-532.
LEMKE, J. J., DURFEE, T. & GOURSE, R. L. 2009. DksA and ppGpp directly regulate transcription of the *Escherichia coli* flagellar cascade. *Molecular Microbiology,* 74**,** 1368-1379.
LI, P., LIU, Q., HUANG, C., ZHAO, X., ROLAND, K. L. & KONG, Q. 2017. Reversible synthesis of colanic acid and O-antigen polysaccharides in *Salmonella* Typhimurium enhances induction of cross-immune responses and provides protection against heterologous *Salmonella* challenge. *Vaccine,* 35**,** 2862-2869.
LIAO, H., ZHONG, X., XU, L., MA, Q., WANG, Y., CAI, Y. & GUO, X. 2019. Quorum-sensing systems trigger catalase expression to reverse the *oxyR* deletion-mediated VBNC state in *Salmonella* typhimurium. *Research in Microbiology,* 170**,** 65-73.
MACNAB, R. M. 1992. Genetics and biogenesis of bacterial flagella. *Annu Rev Genet,* 26**,** 131-58.
MAJDALANI, N. & GOTTESMAN, S. 2005. The Rcs Phosphorelay: A Complex Signal Transduction System. *Annual Review of Microbiology,* 59**,** 379-405.
MALLIK, P., PAUL, B. J., RUTHERFORD, S. T., GOURSE, R. L. & OSUNA, R. 2006. DksA is required for growth phase-dependent regulation, growth rate-dependent control, and stringent control of *fis* expression in *Escherichia coli*. *Journal of bacteriology,* 188**,** 5775-5782.
MARTEYN, B. S., KARIMOVA, G., FENTON, A. K., GAZI, A. D., WEST, N., TOUQUI, L., PREVOST, M. C., BETTON, J. M., POYRAZ, O., LADANT, D., GERDES, K., SANSONETTI, P. J. & TANG, C. M. 2014. ZapE is a novel cell division protein interacting with FtsZ and modulating the Z-ring dynamics. *mBio,* 5**,** e00022-14.
MEGANATHAN, R. & KWON, O. 2009. Biosynthesis of Menaquinone (Vitamin K2) and Ubiquinone (Coenzyme Q). *EcoSal Plus,* 3**,** 10.1128/ecosalplus.3.6.3.3.

MOORE, T. C. & ESCALANTE-SEMERENA, J. C. 2016. The EutQ and EutP proteins are novel acetate kinases involved in ethanolamine catabolism: physiological implications for the function of the ethanolamine metabolosome in *Salmonella enterica*. *Molecular Microbiology,* 99**,** 497-511.
MORONA, R., MANNING, P. A. & REEVES, P. 1983. Identification and characterization of the TolC protein, an outer membrane protein from *Escherichia coli*. *J Bacteriol,* 153**,** 693-9.
NIBA, E. T. E., NAKA, Y., NAGASE, M., MORI, H. & KITAKAWA, M. 2008. A Genome-wide Approach to Identify the Genes Involved in Biofilm Formation in *E. coli*. *DNA Research,* 14**,** 237-246.
NUNN, W. D. & SIMONS, R. W. 1978. Transport of long-chain fatty acids by *Escherichia coli*: mapping and characterization of mutants in the *fadL* gene. *Proc Natl Acad Sci U S A,* 75**,** 3377-81.
OGASAWARA, H., ISHIDA, Y., YAMADA, K., YAMAMOTO, K. & ISHIHAMA, A. 2007. PdhR (pyruvate dehydrogenase complex regulator) controls the respiratory electron transport system in *Escherichia coli*. *Journal of bacteriology,* 189**,** 5534-5541.
PARK, F., GAJIWALA, K., EROSHKINA, G., FURLONG, E., HE, D., BATIYENKO, Y., ROMERO, R., CHRISTOPHER, J., BADGER, J., HENDLE, J., LIN, J., PEAT, T. & BUCHANAN, S. 2004. Crystal structure of YIGZ, a conserved hypothetical protein from *Escherichia coli* k12 with a novel fold. *Proteins: Structure, Function, and Bioinformatics,* 55**,** 775-777.
PFIFFER, V., SARENKO, O., POSSLING, A. & HENGGE, R. 2019. Genetic dissection of *Escherichia coli*'s master diguanylate cyclase DgcE: Role of the N-terminal MASE1 domain and direct signal input from a GTPase partner system. *PLOS Genetics,* 15**,** e1008059.
QIN, R., SANG, Y., REN, J., ZHANG, Q., LI, S., CUI, Z. & YAO, Y.-F. 2016. The Bacterial Two-Hybrid System Uncovers the Involvement of Acetylation in Regulating of Lrp Activity in *Salmonella* Typhimurium. *Frontiers in microbiology,* 7**,** 1864-1864.
RAGHUNATHAN, D., WELLS, T. J., MORRIS, F. C., SHAW, R. K., BOBAT, S., PETERS, S. E., PATERSON, G. K., JENSEN, K. T., LEYTON, D. L., BLAIR, J. M. A., BROWNING, D. F., PRAVIN, J., FLORES-LANGARICA, A., HITCHCOCK, J. R., MORAES, C. T. P., PIAZZA, R. M. F., MASKELL, D. J., WEBBER, M. A., MAY, R. C., MACLENNAN, C. A., PIDDOCK, L. J., CUNNINGHAM, A. F. & HENDERSON, I. R. 2011. SadA, a Trimeric Autotransporter from *Salmonella enterica* Serovar Typhimurium, Can Promote Biofilm Formation and Provides Limited Protection against Infection. *Infection and Immunity,* 79**,** 4342-4352.
REEDER, T. & SCHLEIF, R. 1991. Mapping, sequence, and apparent lack of function of *araJ*, a gene of the *Escherichia coli* arabinose regulon. *J Bacteriol,* 173**,** 7765-71.
ROY, A. & DANCHIN, A. 1982. The *cya* locus of *Escherichia coli* K12: Organization and gene products. *Molecular and General Genetics MGG,* 188**,** 465-471.
SAIER, M. H., JR. & RAMSEIER, T. M. 1996. The catabolite repressor/activator (Cra) protein of enteric bacteria. *J Bacteriol,* 178**,** 3411-7.
SAINI, S., PEARL, J. A. & RAO, C. V. 2009. Role of FimW, FimY, and FimZ in Regulating the Expression of Type I Fimbriae in *Salmonella enterica* Serovar Typhimurium. *Journal of Bacteriology,* 191**,** 3003-3010.
SCHEMBRI, M. A., KJÆRGAARD, K. & KLEMM, P. 2003. Global gene expression in *Escherichia coli* biofilms. *Molecular Microbiology,* 48**,** 253-267.
SCHURR, T., NADIR, E. & MARGALIT, H. 1993. Identification and characterization of *E.coli* ribosomal binding sites by free energy computation. *Nucleic Acids Research,* 21**,** 4019-4023.
SEO, S. W., KIM, D., LATIF, H., O'BRIEN, E. J., SZUBIN, R. & PALSSON, B. O. 2014. Deciphering Fur transcriptional regulatory network highlights its complex role beyond iron metabolism in *Escherichia coli*. *Nature Communications,* 5**,** 4910.
SIRKO, A., ZATYKA, M., SADOWY, E. & HULANICKA, D. 1995. Sulfate and thiosulfate transport in *Escherichia coli* K-12: evidence for a functional overlapping of sulfate- and thiosulfate-binding proteins. *Journal of bacteriology,* 177**,** 4134-4136.
SQUIRES, C. H., DE FELICE, M., WESSLER, S. R. & CALVO, J. M. 1981. Physical characterization of the ilvHI operon of *Escherichia coli* K-12. *J Bacteriol,* 147**,** 797-804.

STENBERG, F., CHOVANEC, P., MASLEN, S. L., ROBINSON, C. V., ILAG, L. L., VON HEIJNE, G. & DALEY, D. O. 2005. Protein complexes of the *Escherichia coli* cell envelope. *J Biol Chem,* 280**,** 34409-19.
SUKHAREV, S. I., BLOUNT, P., MARTINAC, B., BLATTNER, F. R. & KUNG, C. 1994. A large-conductance mechanosensitive channel in *E. coli* encoded by *mscL* alone. *Nature,* 368**,** 265-8.
SZYF, M., AVRAHAM-HAETZNI, K., REIFMAN, A., SHLOMAI, J., KAPLAN, F., OPPENHEIM, A. & RAZIN, A. 1984. DNA methylation pattern is determined by the intracellular level of the methylase. *Proceedings of the National Academy of Sciences of the United States of America,* 81**,** 3278-3282.
TANG, F. & SAIER, M. H. 2014. Transport proteins promoting *Escherichia coli* pathogenesis. *Microbial Pathogenesis,* 71-72**,** 41-55.
TREMBLAY, L. W., DUNAWAY-MARIANO, D. & ALLEN, K. N. 2006. Structure and activity analyses of *Escherichia coli* K-12 NagD provide insight into the evolution of biochemical function in the haloalkanoic acid dehalogenase superfamily. *Biochemistry,* 45**,** 1183-93.
TUCKER, D. L., TUCKER, N., MA, Z., FOSTER, J. W., MIRANDA, R. L., COHEN, P. S. & CONWAY, T. 2003. Genes of the GadX-GadW regulon in *Escherichia coli*. *J Bacteriol,* 185**,** 3190-201.
VALENS, M., THIEL, A. & BOCCARD, F. 2016. The MaoP/*maoS* Site-Specific System Organizes the Ori Region of the *E. coli* Chromosome into a Macrodomain. *PLoS genetics,* 12**,** e1006309-e1006309.
WANG, Z., WANG, J., REN, G., LI, Y. & WANG, X. 2015. Influence of Core Oligosaccharide of Lipopolysaccharide to Outer Membrane Behavior of *Escherichia coli*. *Marine Drugs,* 13**,** 3325-3339.
WINSTON, F., BOTSTEIN, D. & MILLER, J. H. 1979. Characterization of amber and ochre suppressors in *Salmonella* typhimurium. *Journal of Bacteriology,* 137**,** 433-439.
WU, X., HOU, J., CHEN, X., CHEN, X. & ZHAO, W. 2016. Identification and functional analysis of the L-ascorbate-specific enzyme II complex of the phosphotransferase system in *Streptococcus mutans*. *BMC Microbiology,* 16**,** 51.
YEOM, J., GAO, X. & GROISMAN, E. A. 2018. Reduction in adaptor amounts establishes degradation hierarchy among protease substrates. *Proceedings of the National Academy of Sciences,* 115**,** E4483-E4492.
YEW, W. S. & GERLT, J. A. 2002. Utilization of L-ascorbate by *Escherichia coli* K-12: assignments of functions to products of the *yjf-sga* and *yia-sgb* operons. *Journal of bacteriology,* 184**,** 302-306.
ZHANG, Y., MORAR, M. & EALICK, S. E. 2008. Structural biology of the purine biosynthetic pathway. *Cellular and Molecular Life Sciences,* 65**,** 3699-3724.
